# Population genetics and genetic variation of *Ctenocephalides felis* and *Pulex irritans* in China by analysis of nuclear and mitochondrial genes

**DOI:** 10.1186/s13071-022-05393-6

**Published:** 2022-07-27

**Authors:** Yu Zhang, Yu Nie, Le-Yan Li, Shu-Yu Chen, Guo-Hua Liu, Wei Liu

**Affiliations:** 1 Research Center for Parasites & Vectors, College of Veterinary Medicine, Hunan Agricultural University, Changsha, 410128 Hunan China; 2 The Key Laboratory of Animal Vaccine & Protein Engineering, Changsha, 410128 Hunan China

**Keywords:** *Ctenocephalides felis*, *Pulex irritans*, Population genetics, Evolutionary biology, ITS1, EF-1α, *cox*1, *cox*2

## Abstract

**Background:**

Fleas are the most economically significant blood-feeding ectoparasites worldwide. *Ctenocephalides felis* and *Pulex irritans* can parasitize various animals closely related to humans and are of high veterinary significance.

**Methods:**

In this study, 82 samples were collected from 7 provinces of China. Through studying the nuclear genes ITS1 and EF-1α and two different mitochondrial genes *cox*1 and *cox*2, the population genetics and genetic variation of *C. felis* and *P. irritans* in China were further investigated.

**Results:**

The intraspecies differences between *C. felis* and *P. irritans* ranged from 0 to 3.9%. The interspecific variance in the EF-1α, *cox*1, and *cox*2 sequences was 8.2–18.3%, while the ITS1 sequence was 50.1–52.2%. High genetic diversity was observed in both *C. felis* and *P. irritans*, and the nucleotide diversity of *cox*1 was higher than that of *cox*2. Moderate gene flow was detected in the *C. felis* and *P. irritans* populations. Both species possessed many haplotypes, but the haplotype distribution was uneven. Fu's Fs and Tajima's D tests showed that *C. felis* and *P. irritans* experienced a bottleneck effect in Guangxi Zhuang Autonomous Region and Henan province. Evolutionary analysis suggested that *C. felis* may have two geographical lineages in China, while no multiple lineages of *P.irritans* were found.

**Conclusions:**

Using sequence comparison and the construction of phylogenetic trees, we found a moderate amount of gene flow in the *C. felis* and *P. irritans* populations. Both species possessed many haplotypes, but the distribution of haplotypes varied among the provinces. Fu’s Fs and Tajima’s D tests indicated that both species had experienced a bottleneck effect in Guangxi and Henan provinces. Evolutionary analysis suggested that *C. felis* may have two geographical lineages in China, while no multiple lineages of *P.irritans* were found. This study will help better understand fleas' population genetics and evolutionary biology.

**Graphical Abstract:**

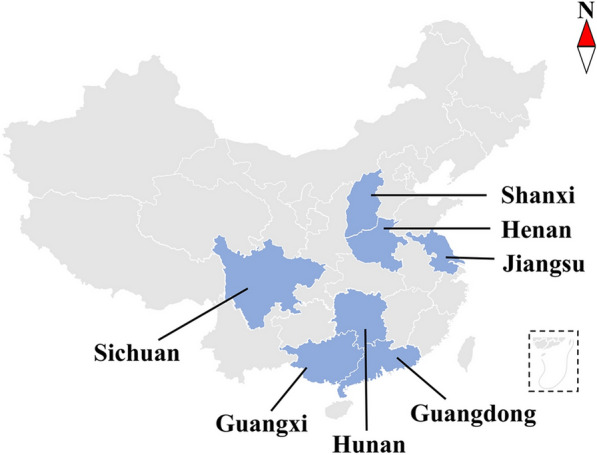

## Background

Fleas are the most economically significant blood-feeding ectoparasites worldwide. They account for 50% of skin diseases in cats and dogs at veterinary clinics [1], and > $15 billion is spent worldwide to control flea infections in companion animals [2]. Fleas are vectors for diseases such as bartonellosis, murine typhus, cat scratch disease, and the bubonic plague [3–7]. The cat flea *Ctenocephalides felis* and the human flea *Pulex irritans* are characterized by various hosts, including humans, carnivores, rodents, and ungulates [8, 9] and exhibit high genetic diversity and complex genetic structure. *C. felis* and *P. irritans* are closely related to human and animal life and are, therefore, of high veterinary significance [8, 10]. Understanding fleas' vector transmission and disease epidemiology will help develop strategies to prevent and control them [11, 12].

With the development of genetic technologies, which complement, to some extent, the limitations of traditional morphology, molecular biology methods have been widely used in taxonomy, systematics, and population genetics, including those of fleas [13, 14]. The genetic diversity of fleas has also been studied using nuclear markers, such as histone H3, EF-1α, ITS1, and ITS2 [13, 15–17]. Mitochondria caught the attention of evolutionary biologists in the 1960s because of their small size, high abundance in cells, and simple mode of inheritance [18, 19]. The rapid mutation rates of mtDNA compared with nDNA facilitates the analysis of recent divergences within and between species [20]. The mtDNA markers cytochrome c oxidase subunits 1 and 2, named *cox*1 and *cox*2, are frequently used to evaluate genetic diversity and identify cryptic species and the population structure of invertebrates [10, 21–23]. Van der Mescht et al. suggested in 2015 that host specificity may influence the level of intraspecies genetic differentiation [24]. Hornok et al. investigated this hypothesis in 2018 and found large differences in mitochondrial sequences in some synanthropic flea species, such as *C. felis* and *P. irritans* [25].

The genetic diversity of *C. felis* has so far been investigated in Africa [13], the USA [26, 27], Europe [25], Asia [28, 29], and Australia-New Zealand [10, 30, 31]. In 2020, Azrizal-Wahid et al. used cytochrome *c* oxidase subunit I (*cox*1) and II (*cox*2) to study the genetic lineages of the *C. felis* population in Malaysia and revealed two main lineages, with Malaysian haplotypes closely related to tropical haplotypes [12].

In 2019, Zurita et al. studied the classification, origin, evolution, and phylogeny of *P. irritans* from two populations in Spain and Argentina, using the internal transcribed spacer (ITS)1 and ITS2 and partial cytochrome *c* oxidase subunit 1 (*cox*1) and cytochrome *b* (*cyt*b) genes. The study found a considerable degree of molecular differentiation between the two populations and found two clear geographic genetic lineages, indicating the presence of two cryptic species [17].

Fleas have previously been studied in parts of China [29, 32], but on the Chinese mainland, there have been no studies into the intraspecific genetic diversity, evolutionary relationships, or transmission patterns of *C. felis* and *P. irritans*. In this study, two nDNA markers, ITS1 and EF-1α, were combined with two different mtDNA markers, *cox*1 and *cox*2, to (i) provide detailed information that expands the knowledge of the genetic diversity of *C. felis* and *P. irritans* in various provinces of China; (ii) provide a basis for the re-evaluation of the population structure and genetic differentiation of *C. felis* and *P. irritans* in China; and (iii) infer phylogenetic relationships from the geographical distribution of different isolates.

## Methods

### Parasites and DNA extraction

**Fig. 1:**
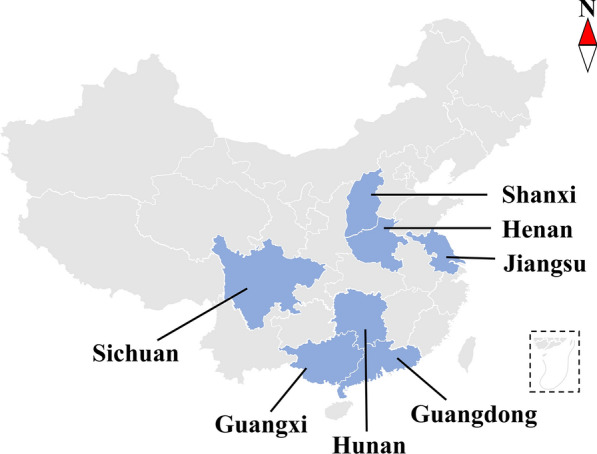
The geographic distribution of fleas in this study

A total of 82 fleas were obtained from different regions of China (Fig. 1). The fleas were observed and photographed with a stereomicroscope, and the features of the shape of the frons, mane, comb, and other parts were used for preliminary identification [10, 13, 25, 33–35]. All fleas were supplied in ≥ 70% (v/v) ethanol and stored at − 20 °C. The whole specimen was used to extract DNA. According to the specification; total genomic DNA was isolated using sodium dodecyl sulfate/proteinase K treatment, followed by spin-column purification (Wizard^®^ SV Genomic DNA Purification System; Promega Madison, WI, USA) from the manufacturer. The sample codes, host, locality, and GenBank accession numbers are listed in Table 1.

**Table 1:** GenBank accession numbers of *C. felis* and *P. irritans* from China

Species	Sample codes	Locality	GenBank accession number			
			ITS1	EF-1α	*cox*1	*cox*2
*C.felis*	HUN1-HUN11	Hunan	ON113962-ON11396; ON113981; ON114001-ON114004; ON114034-ON114036	ON561116-ON561117	ON398417-ON398418; ON398481-ON398482; ON398699-ON398702; ON398707-ON398709	ON508801-ON508805
	GD1-GD8	Guangdong	ON114005-ON114007; ON113996; ON114027-ON114030	ON561118-ON561122	ON399381-ON399388	ON508806-ON508811
	HN1-HN11	Henan	ON113995; ON114008-ON114014; ON114031-ON114033	ON561128-ON561131	ON399054-ON399064	ON508812-ON508815
	JS1-JS10	Jiangsu	ON113982-ON113989; ON114015-ON114016	ON561113-ON561115	ON399065-ON399074	ON561111-ON561112
	SX1-SX9	Shanxi	ON113990-ON113994; ON114017-ON114018; ON114025-ON114026	ON561123-ON561127	ON399190-ON399198	ON508816-ON508819
	GX1-GX10	Guangxi	ON113997-ON114000; ON114019-ON114024	ON569077-ON569081	ON399199-ON399208	ON508820-ON508824
*P. irritans*	SC1-SC10	Sichuan	ON114050-ON114059	ON569082-ON569083	ON406172-ON406181	ON561108-ON561110
	PHN1-PHN13	Henan	ON114037- ON114049	ON569084-ON569087	ON406182-ON406194	ON455233-ON455234

### Enzymatic amplification and sequencing

ITS1 was amplified with the primers Sen-ITS1 (5ʹ-GTACACACCGCCCGTGCGTACT-3ʹ) and Rev-ITS1 (5ʹ-GCT GCGTTCTTCATCGACCC-3ʹ) [15]. The cycling was as follows: initial denaturing at 94 °C for 5 min followed by 35 cycles of 94 °C for 30 s, 58 °C for 30 s, 72 °C for 90 s, and a final elongation for 5 min at 72 °C. EF-1α was amplified using EF-1α-F (5ʹ-AATTGAAGGCCGAACGTGAG-3ʹ) and EF-1α-R (5ʹ-GATTTGCCAGTACGACGGTC-3ʹ) [13]. The cycling was as follows: initial denaturing at 95 °C for 1 min followed by 35 cycles of 95 °C for 15 s, 55 °C for 15 s, 72 °C for 10 s, and a final elongation for 5 min at 72 °C. *cox*1 was amplified using LCO1490 (5ʹ-GGTCAACAAATCATAAAGATATTGG-3ʹ) [36] and Cff-R [S0368] (5ʹ-GAAGGGTCAAAGAATGATGT-3ʹ) [10]. The cycling was as follows: initial denaturing at 95 °C for 1 min followed by 35 cycles of 95 °C for 15 s, 49 °C for 15 s, 72 °C for 10 s, and a final elongation for 5 min at 72 °C. *cox*2 was amplified using F-Leu (5ʹ-TCTAATATGGCAGATTAGTGC-3ʹ) and R-Lys (5ʹ-GAGACCAGTACTTGCTTT CAGTCATC-3ʹ) [37]. The cycling was as follows: initial denaturing at 95 °C for 3 min followed by 37 cycles of 94 °C for 30 s, 42 °C for 30 s, 72 °C for 15 s, and a final elongation for 5 min at 72 °C.

All PCR reactions (25 μl) contained 1 µl of DNA sample, 0.5 μl of each primer, 12.5 μl of 2 × Premix EmeraldAmp Max HS PCR Master Mix (TaKaRa, Dalian, China), and 10.5 μl of ddH_2_O. All PCRs were run on a thermocycler (Bio-Rad, Hercules, CA, USA). The results were validated by electrophoresis on 1% agarose gels. PCR products were bi-directionally sequenced by Sangon Biotech Co., Ltd. (Shanghai, China). BLAST analysis was performed on the sequencing results of four genes of all samples to further determine the species of flea collected.

### Population differentiation

All  ITS1, EF-1α, *cox*1, and *cox*2 sequences were compared using Clustal X 1.83 software and then manually cut. MegAlign software was used to analyze the sequence differences among four genes [38]. The DnaSP 5.0 program was used to determine the haplotypes, nucleotide diversity (Pi), and haplotype diversity (Hd) of each gene [39]. DnaSP was also used to calculate the correlation among geographic locations, genetic differentiation index (Fst), and the corresponding gene flow (Nm) between populations [12]. For the *cox*1, *cox*2, and concatenated sequences, DnaSP 5.0 was used to calculate Tajima's D [40] and Fu’s Fs [41] statistics to study the population history of *C. felis* in six regions and *P. irritans* in two regions. DnaSP 5.0 and PopART 1.7 were used to create a statistically parsimonious network to infer haplotype relationships [42].

### Sequences analysis and reconstruction of phylogenetic relationships

The EF-1α sequence and the tandem sequence of *cox*1 + *cox*2 of *C. felis* and *P. irritans* from China were compared and analyzed. For the EF-1α sequence, *Megabothris calcarifer* was used as the outgroup, and for *cox*1 + *cox*2, *Ceratophyllus wui* was used as the outgroup.

Clustal X 1.83 was used to align the sequences of these four genes separately [38], and then the EF-1α sequences and the concatenated sequences of *cox*1 and *cox*2 were compared using Gblock 0.91 [43]. Using MEGA X, the phylogenetic trees were constructed using the maximum likelihood method [44]. Relative support for the tree topology was obtained by bootstrapping using 1000 replicates.

## Results

### Analysis of genetic diversity

A total of 82 samples (59 *C. felis* and 23 *P. irritans*) from seven provinces in China (Table 1) were analyzed. ITS1, EF-1α, *cox*1, and *cox*2 fragments (761, 771, 491, and 571 bp, respectively) were amplified and uploaded to GenBank.

The ITS1, EF-1α, *cox*1, and *cox*2 sequences were compared. ITS1 sequence comparison showed that the intraspecific difference of *C. felis* was 0–0.7%, that of *P. irritans* was 0–3.8%, and the interspecific difference between *C. felis* and *P. irritans* was 50.1–52.2%. The results of the EF-1α sequence comparison showed that the intraspecific differences between *C. felis* and *P. irritans* were 0–1.6% and 0–1.2%, respectively, and the interspecific difference between *C. felis* and *P. irritans* was 8.2–10%. The *cox*1 sequence analysis showed that the intraspecific difference of *C. felis* was 0–3.9%, that of *P. irritans* was 0–3.6%, and the interspecific difference between *C. felis* and *P. irritans* was 13.3–17.9%. The *cox*2 sequence analysis showed that the intraspecific difference of *C. felis* was 0–1.9%, that of *P. irritans* 0–0.5%, and the interspecific difference between *C. felis* and *P. irritans* was 17.1–18.3%.

For *C. felis*, the Hd and Pi were 0.834 and 0.18944, respectively, according to *cox*1 sequence analysis. The Hd was 0.819, and the Pi was 0.02544, according to cox2 sequencing analysis. In tandem sequence analysis, the Hd was 0.901, and the Pi was 0.09145 (Table 2). In the overall mean data, the *cox*1 gene showed higher Hd than the *cox*2 gene, while for the Pi, *cox*1 showed higher genetic diversity than *cox*2. For *P. irritans*, Hd and Pi were 0.917 and 0.07117, respectively, according to *cox*1 sequence analysis. In *cox*2 sequence analysis, Hd and Pi were 0.889 and 0.45925, respectively. The results of tandem sequence analysis indicated that the Hd and Pi were 0.9763 and 0.29144, respectively (Table 2). The Hd of the *cox*1 gene was higher than that of the *cox*2 gene, while the genetic diversity of Pi of the *cox*1 gene was lower than that of the *cox*2 gene.

**Table 2:** Haplotype, nucleotide diversity, haplotype diversity, Tajima’s D, and Fu’s Fs based on *cox*1, *cox*2, and concatenated *cox*1 + *cox*2 of *C. felis* and *P.irritans* from China

Species	Gene	Region	Hp	Pi	Hd	Tajima’s D	Fu’s Fs
*C. felis*	*cox*1	Hunan	3	0.00642	0.618	1.43721 (*P* > 0.10)	3.592 (*P* > 0.10)
		Guangdong	3	0.00701	0.607	− 0.02190 (*P* > 0.10)	2.6 (*P* > 0.10)
		Henan	11	0.6754	1.0	− 1.02038 (*P* > 0.10)	1.722 (*P* > 0.10)
		Jiangsu	7	0.00477	0.911	0.21500 (*P* > 0.10)	− 2.460 (*P* > 0.10)
		Shanxi	3	0.00690	0.417	− 0.36752 (*P* > 0.10)	3.189 (*P* > 0.10)
		Guangxi	3	0.00302	0.378	*− 1.87333	*1.453
		Totally	17	0.18944	0.834	**− 2.30995	31.324 (*P* > 0.10)
	*cox*2	Hunan	2	0.00291	0.509	1.68091 (*P* > 0.10)	3.360 (*P* > 0.10)
		Guangdong	4	0.00358	0.821	0.01486 (*P* > 0.10)	0.459 (*P* > 0.10)
		Henan	4	0.09344	0.673	**− 2.06809	*17.368
		Jiangsu	2	0.00030	0.200	− 1.11173 (*P* > 0.10)	− 0.339 (*P* > 0.10)
		Shanxi	3	0.00432	0.417	− 1.66774 (0.1 > *P* > 0.05)	2.455 (0.1 > *P* > 0.05)
		Guangxi	1	0	0	–	–
		Totally	8	0.02544	0.819	***− 2.78207	*14.949
	*cox*1 + *cox*2	Hunan	3	0.00463	0.618	1.68254 (*P* > 0.10)	5.048 (*P* > 0.10)
		Guangdong	4	0.00596	0.821	− 0.00622 (*P* > 0.10)	2.393 (*P* > 0.10)
		Henan	11	0.34293	1	− 1.23462 (*P* > 0.10)	1.902 (*P* > 0.10)
		Jiangsu	8	0.00235	0.956	− 0.04254 (*P* > 0.10)	− 3.796 (*P* > 0.10)
		Shanxi	3	0.00547	0.417	− 1.10200 (*P* > 0.10)	5.150 (*P* > 0.10)
		Guangxi	3	0.00140	0.378	*− 1.87333	*1.453
		Totally	21	0.09145	0.901	**− 2.43360	*27.356
*P.irritans*	*cox*1	Henan	12	0.12198	0.987	***− 2.33196	*0.845
		Sichuan	6	0.00583	0.778	*− 1.86483	*− 0.695
		Totally	17	0.07117	0.917	***− 2.69698	*1.257
	*cox*2	Henan	11	0.61,732	0.974	− 0.57701 (*P* > 0.10)	7.243 (*P* > 0.10)
		Sichuan	3	0.00113	0.644	0.22171 (*P* > 0.10)	− 0.046 (*P* > 0.10)
		Totally	13	0.45925	0.889	− 0.92245 (*P* > 0.10)	24.165 (*P* > 0.10)
	*cox*1 + *cox*2	Henan	13	0.39918	1	− 0.95776 (*P* > 0.10)	1.718 (*P* > 0.10)
		Sichuan	7	0.00324	0.867	− 1.62353 (0.1 > *P* > 0.05)	− 1.343 (0.1 > *P* > 0.05)
		Totally	20	0.29144	0.9763	− 1.30410 (*P* > 0.10)	5.307(*P* > 0.10)

*Hp* number of haplotypes, *Pi* nucleotide diversity, *Hd* haplotype diversity

^*^Significant *P* value < 0.05 (*P* < 0.05)

^**^Significant *P* value < 0.01 (*P* < 0.01)

^***^Significant *P* value < 0.001 (*P* < 0.001)

According to the known classification criteria, Fst > 0.25, is defined as great differentiation; 0.15 < Fst < 0.25 is defined as moderate differentiation, and Fst < 0.15 is defined as a trivial difference [45]. According to Slatkin [46], Nm > 1 indicates high gene flow; 0.25 < Nm < 0.99 is intermediate gene flow; and Nm < 0.25 is classified as low gene flow. For *C. felis,* Fst, and Nm among the six regions are shown in Table 3. The overall Fst and Nm of all samples were 0.34897 and 0.93, respectively, indicating great genetic differentiation and intermediate gene flow of *C. felis* in China. Guangxi Zhuang Autonomous Region and Jiangsu province had the highest Fst (0.89152), while Guangdong and Shanxi provinces had the lowest Fst (− 0.05614). The Fst value of population pairs in Guangdong and Shanxi provinces was not significantly different, and the Fst values of population pairs in Hunan and Guangdong provinces and Hunan and Shanxi provinces were moderate, while the Fst values of population pairs in other regions were significantly different. Among the six sampling regions, the gene flow was the largest in Guangdong and Hunan provinces (1.66) and the smallest in Shanxi and Guangdong provinces (− 9.41). Low levels of gene flow were found between Guangxi Zhuang Autonomous Region and Hunan province, Guangxi Zhuang Autonomous Region and Guangdong province, Jiangsu province and Guangxi Zhuang Autonomous Region, Shanxi and Guangdong provinces, and Shanxi province and Guangxi Zhuang Autonomous Region. Medium levels of gene flow were found between Guangxi Zhuang Autonomous Region and Henan province, Guangdong and Jiangsu provinces, Hunan and Jiangsu provinces, and Shanxi and Jiangsu provinces. High gene flow was found between the other regions. For *P. irritans*, the level of genetic differentiation between Henan province and Sichuan province was high (Fst = 0.37433), and the level of gene flow was intermediate (Nm = 0.84).

**Table 3:** Pairwise genetic differentiation (Fst: below diagonal) and gene flow (Nm: above diagonal) among *C. felis* populations in six regions in China

Region	Hunan	Guangdong	Henan	Guangxi	Jiangsu	Shanxi
Hunan	–	1.66	1.05	0.09	0.37	1.50
Guangdong	0.23201	–	1.10	0.11	0.66	−9.41
Henan	0.32185	0.31156	–	0.98	1.08	1.10
Guangxi	0.85405	0.81962	0.33834	–	0.06	0.14
Jiangsu	0.57690	0.43237	0.31660	0.89152	–	0.58
Shanxi	0.24954	-0.05614	0.31209	0.78640	0.46465	–

### Population expansion

In the existing *C. felis*, the Fu’s F neutrality test of *cox*1, *cox*2, and the serial sequence were positive, except for the *cox*2 sequence in Guangxi Zhuang Autonomous Region, and *cox*1, *cox*2, and the serial sequence in Jiangsu province. In Tajima's D neutrality test, *cox*1 from Jiangsu province, *cox*2 from Guangdong province, and *cox*1, *cox*2, and their serial sequences from Hunan province were all positive, while *cox*2 from Guangxi Zhuang Autonomous Region had no Tajima’s D neutrality test value, and the rest were negative. There were no statistically significant differences except for *cox*1 in Henan province and *c*ox1 and the serial sequence in Guangxi Zhuang Autonomous Region. The significance of the neutrality test in Guangxi Zhuang Autonomous Region is a sign of population balance. *cox*2 in Henan province and *cox*1 and the tandem sequence in Guangxi Zhuang Autonomous Region were positive, indicating that these two regions' populations shrank dramatically because of equilibrium selection and bottleneck effects (Table 2).

For *P. irritans*, Fu’s F neutrality test was positive except for *cox*1, *cox*2, and the serial sequence from Sichuan province. In the neutrality test of Tajima’s D, *cox*2 from Sichuan province was positive, and the rest were negative. Only the *cox*1 test values of these two regions had statistical significance, among which the population neutral test value of Henan province was significant, a sign of population balance.

### Haplotype sequence analysis

The haplotype distribution of *cox*1, *cox*2, and the concatenated *cox*1 + *cox*2 of *C. felis* and *P. irritans* are shown in Table 4. A total of 17 haplotypes were identified for *cox*1 (A1-17), 8 haplotypes for *cox*2 (B1-8), and 21 haplotypes for *cox*1 + *cox*2 (AB1-21) of *C. felis*. The *cox*1 haplotype A1 (*n* = 21) was the most common, followed by A4 (*n* = 9), and the least common types of haplotypes were A6, A7, A8, A9, A10, A11, A12, A13, A15, A16, and A17. In *cox*2, the most common haplotype was B4 (*n* = 16), followed by B1 (*n* = 14), and the least common haplotypes were B6 and B8. In the *cox*1 + *cox*2 series, haplotype AB1 (*n* = 14) was the most common, followed by AB5 (*n* = 9), and the least common haplotypes were AB4, AB6, AB8, AB9, AB12, AB13, AB14, AB15, AB16, AB17, AB18, AB19, AB20, and AB21.

**Table 4:** Haplotype distribution of *C. felis* and *P.irritans* from China based on *cox*1, *cox*2, and concatenated *cox*1 + *cox*2 genes corresponding to the seven sampling locations. The number in parentheses refers to the relative frequency of the given haplotype

Species	Region	No. of flea (*n*)	Haplotype		
			*cox*1	*cox*2	*cox*1 + *cox*2
*C. felis*	Hunan	11	A1(4), A14(6), A15(1)	B1(4), B7(7)	AB1(4), AB7(6), AB8(1)
	Guangdong	8	A1(5), A2(2), A3(1)	B1(3), B2(2), B3(2), B4(1)	AB1(3), AB2(2), AB3(2), AB4(1)
	Henan	11	A2(1), A5(1), A6(1), A7(1), A8(1), A9(1), A10(1), A11(1), A12(1), A13(1),	B3(4), B4(6), B6(1)	AB2(1), AB10(2), AB14(1), AB15(1), AB16(1), AB17(1), AB18(1), AB19(1), AB20(1), AB21(1)
	Guangxi	10	A3(1), A4(9)	B5(10)	AB5(9), AB6(1)
	Jiangsu	10	A1(5), A5(4), A16(1)	B4(9), B8(1)	AB9(1), AB10(3), AB11(5), AB12(1)
	Shanxi	9	A1(7), A2(1), A17(1)	B1(7), B3(1), B5(1)	AB1(7), AB2(1), AB13(1)
*P.irritans*	Sichuan	10	C1(5), C13(1), C14(1), C15(1), C16(1), C17(1)	D1(5), D2(4), D3(1)	CD1(4), CD2(1), CD3(1), CD4(1), CD5(1), CD6(1), CD7(1)
	Henan	13	C1(2), C2(1), C3(1), C4(1), C5(1), C6(1), C7(1), C8(1), C9(1), C10(1), C11(1), C12(1)	D1(2), D4(1), D5(2), D6(1), D7(1), D8(1), D9(1), D10(1), D11(1), D12(1), D13(1)	CD8(1), CD9(1), CD10(1), CD11(1), CD12(1), CD13(1), CD14(1), CD15(1), CD16(1), CD17(1), CD18(1), CD19(1), CD20(1)

For *P. irritans,* there were 17 haplotypes for *cox*1 (C1-17), 13 haplotypes for *cox*2 (D1-13), and 20 haplotypes for *cox*1 + *cox*2 (CD1-20). In *cox*1, haplotype C1 (*n* = 7) was the most common, while in *cox*2, haplotype D1 (*n* = 7) was the most common, followed by haplotype D2 (*n* = 4) and haplotype D5 (*n* = 2). In the *cox*1 + *cox*2 series, haplotype CD1 (*n* = 4) was the most common, and all the other haplotypes had one sample.

### Network analysis

**Fig. 2:**
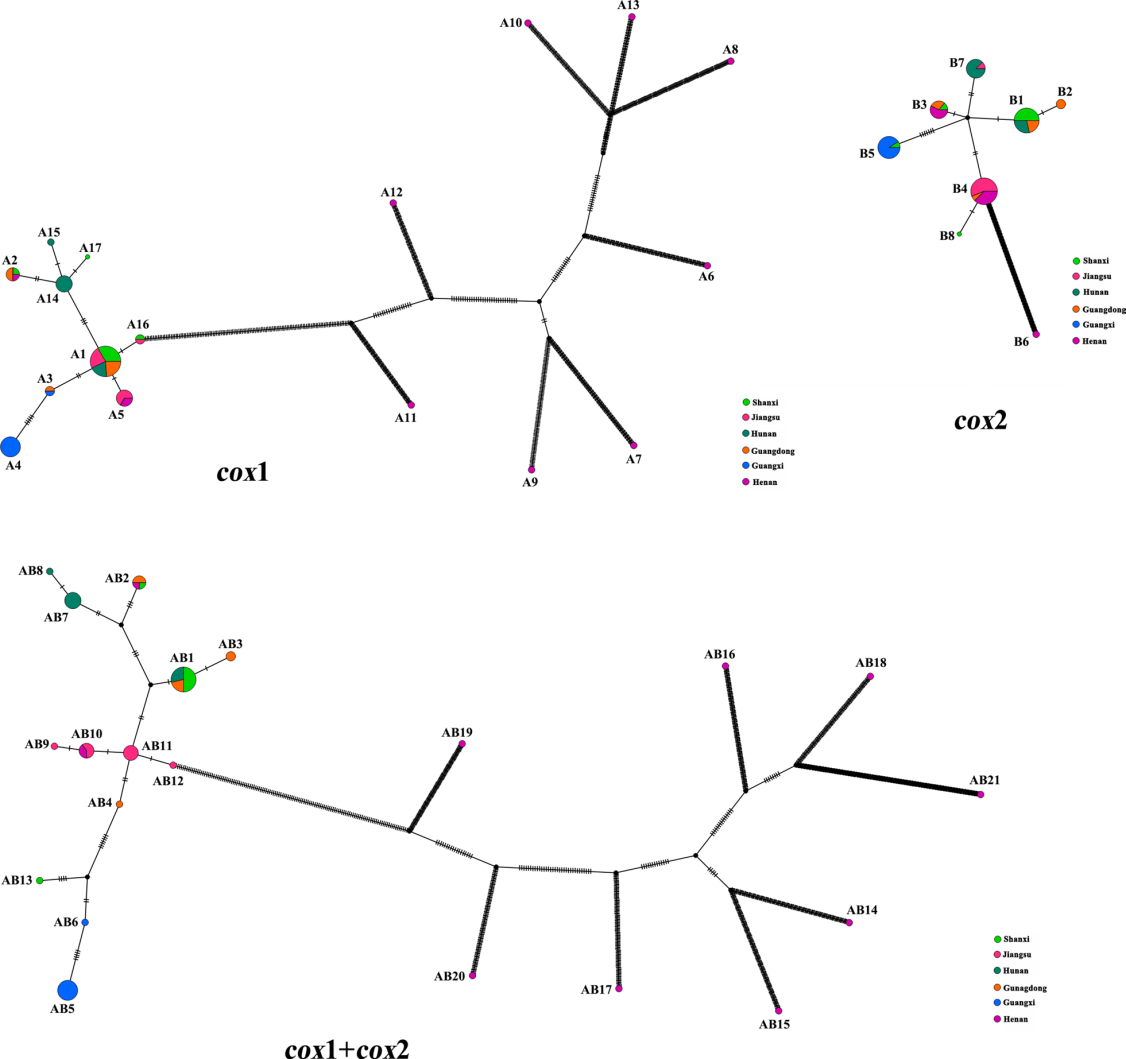
Network maps of *cox*1, *cox*2, and the concatenated sequence haplotypes of *C.felis*

**Fig. 3:**
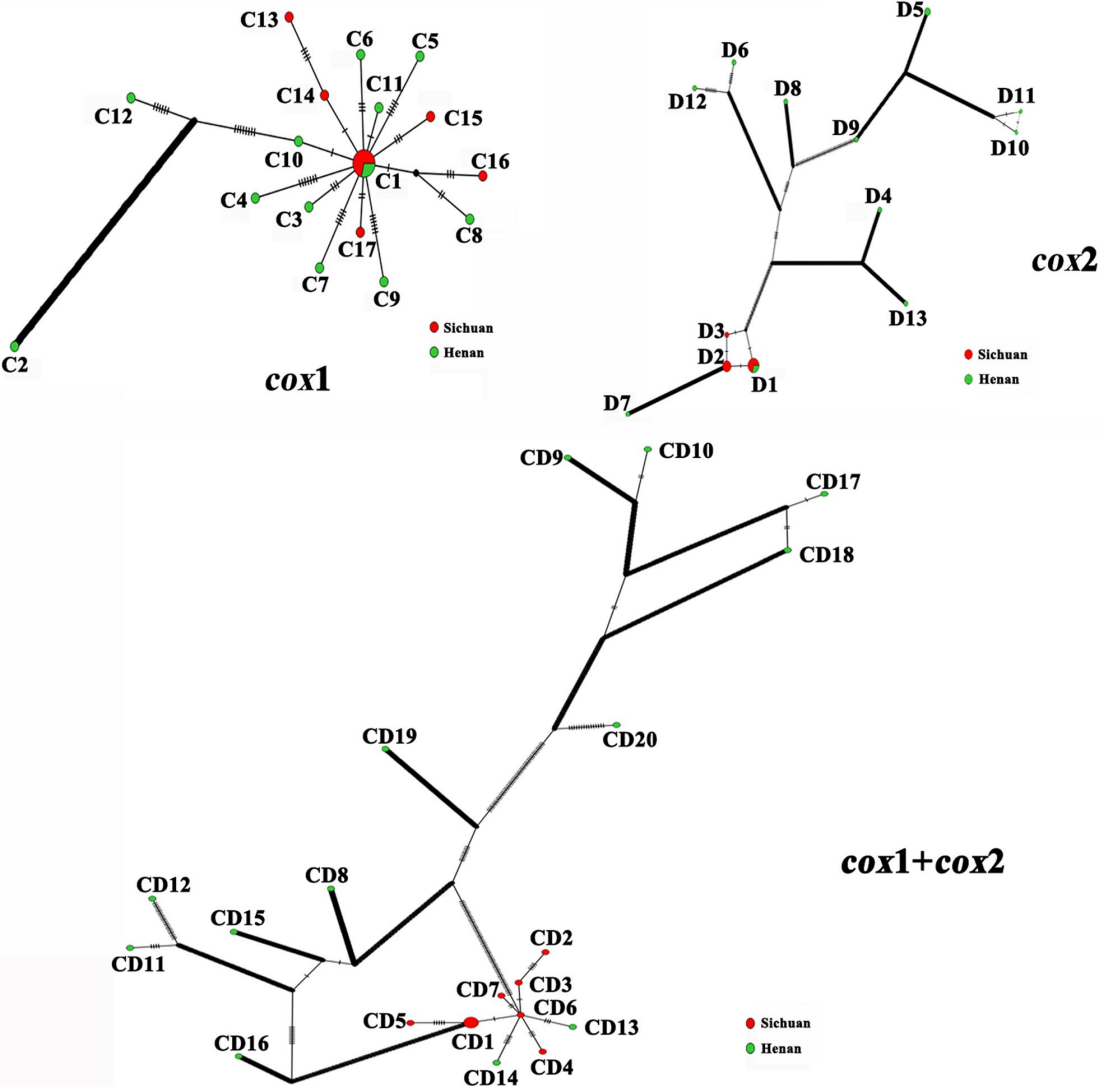
Network maps of *cox*1, *cox*2, and the concatenated sequence haplotypes of *P.irritans*

The complete network of haplotypes of *C. felis* and *P. irritans* was constructed (Figs. 2 and 3). In *C. felis*, 17 *cox*1 haplotypes (A1-17) were distributed in a star shape around the A1 haplotype, which contained 21 individuals, including 4 from Hunan province, 5 from Guangdong province, 5 from Jiangsu province, and 7 from Shanxi province. Henan province had the largest haplotypes, with ten haplotypes (A2, A5, A6, A7, A8, A9, A10, A11, A12, and A13), while Guangxi Zhuang Autonomous Region had the fewest haplotypes (A3 and A4). The A4 haplotype was found only in Guangxi Zhuang Autonomous Region, the A6-A13 haplotypes were found only in Henan province, the A14 and A15 haplotypes were found only in Hunan province, the A16 haplotype was found only in Jiangsu province, and the A17 haplotype was found only in Shanxi province (Fig. 2, Table 4). Compared with *cox*1, *cox*2 haplotypes (B1–B8) were fewer and less widely distributed (Fig. 2). Guangdong province had the most haplotypes (B1–B4), while Guangxi Zhuang Autonomous Region had only one haplotype, B5. The B2 haplotype was found only in Guangdong province, the B6 haplotype was found only in Henan province, and the B8 haplotype was found only in Jiangsu province. Among the 21 haplotypes generated by the sequence data of the *cox*1 + *cox*2 series, AB11 was at the center of the remaining 20 haplotypes, forming a star pattern (Fig. 2). There were ten haplotypes (AB2, AB10, AB14, AB15, AB16, AB17, AB18, AB19, AB20, and AB21) in Henan province and two haplotypes (AB5 and AB64) in Guangxi Zhuang Autonomous Region, results which were the same as those of the *cox*1 haplotype. Haplotypes AB3 and AB4 were found only in Guangdong province, the AB5 and AB6 haplotypes were found only in Guangxi Zhuang Autonomous Region, the AB14-21 haplotypes were found only in Henan province, the AB7 and AB8 haplotypes were found only in Hunan province, the AB9, AB11, and AB12 haplotypes were found only in Jiangsu province, and the AB13 haplotype was found only in Shanxi province. In the populations of *C. felis*, the most common haplotypes were A1 of *cox*1, B1 of *cox*2, and AB1 of *cox*1 + *cox*2 (Fig. 2).

In the *cox*1 network of *P. irritans*, 16 haplotypes (C2-17) formed a star shape with the C1 haplotype as the center. C1 was the only haplotype that appeared in both Sichuan and Henan provinces. The C2-12 haplotypes were found only in Henan province, and the C13-17 haplotypes were found only in Sichuan province (Fig. 3, Table 4). Among the 13 haplotypes of *cox*2 (D1-13), the D2 and D3 haplotypes were found only in Sichuan province, and the D4-13 haplotypes were found only in Henan province, except D1 was found in both places (Fig. 3, Table 4). Twenty haplotypes (CD1-20) were generated from the *cox*1 + *cox*2 series data. The CD1-7 haplotypes were found only in Sichuan province, and the CD8-20 haplotypes were found only in Henan province (Fig. 3, Table 4). In the populations of *P. irritans*, the most common haplotypes were C1 of *cox*1, D1 of *cox*2, and CD1 of *cox*1 + *cox*2 (Fig. 3).

### Phylogenetic analysis

To study the phylogenetic relationships among *C. felis* and *P. irritans* isolates from different regions in China, we analyzed sequences of EF-1α and the tandem sequences of the *cox*1 and *cox*2 genes and constructed phylogenetic trees.

**Fig. 4:**
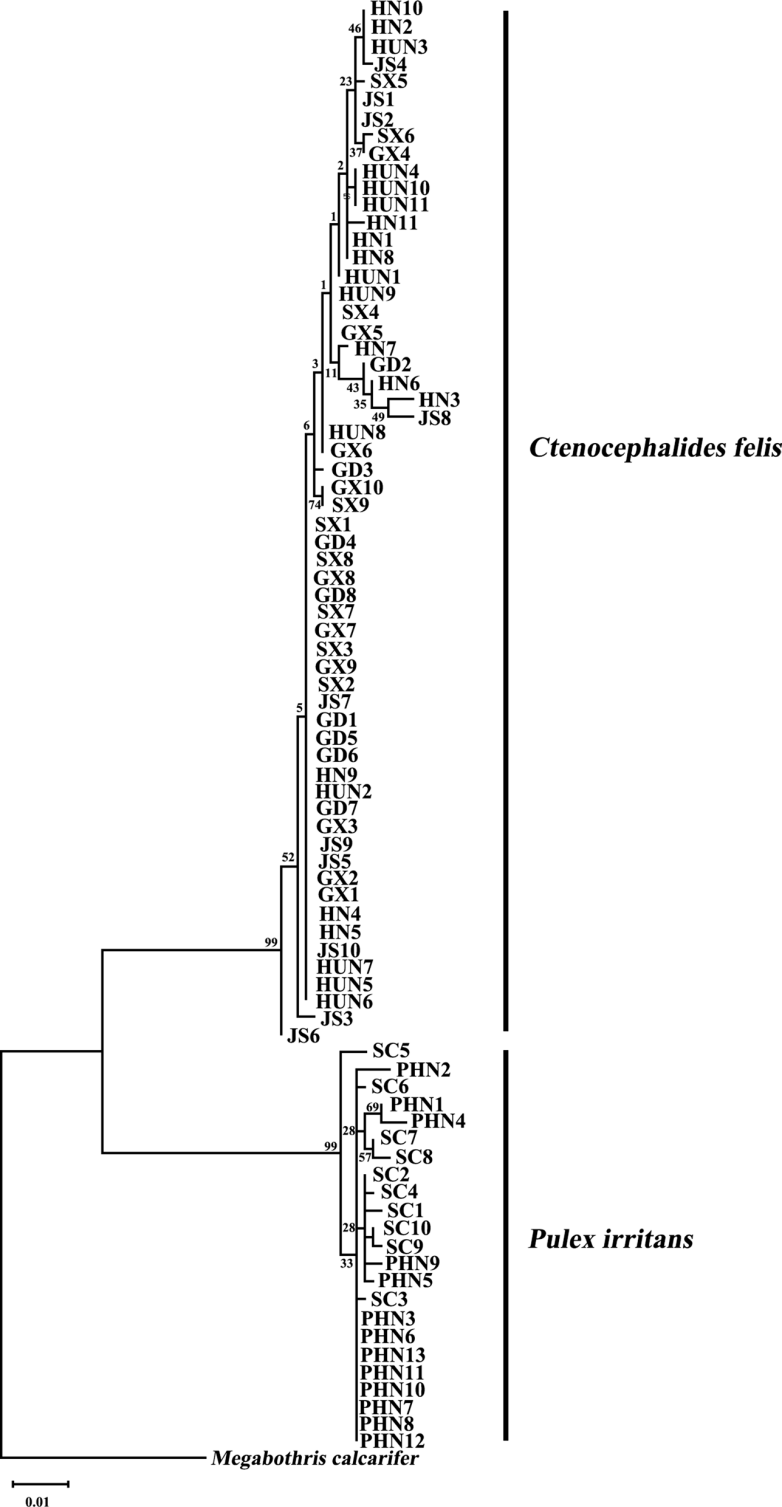
Phylogenetic relationships between *C.felis* and *P.irritans* based on EF-1α sequences

**Fig. 5:**
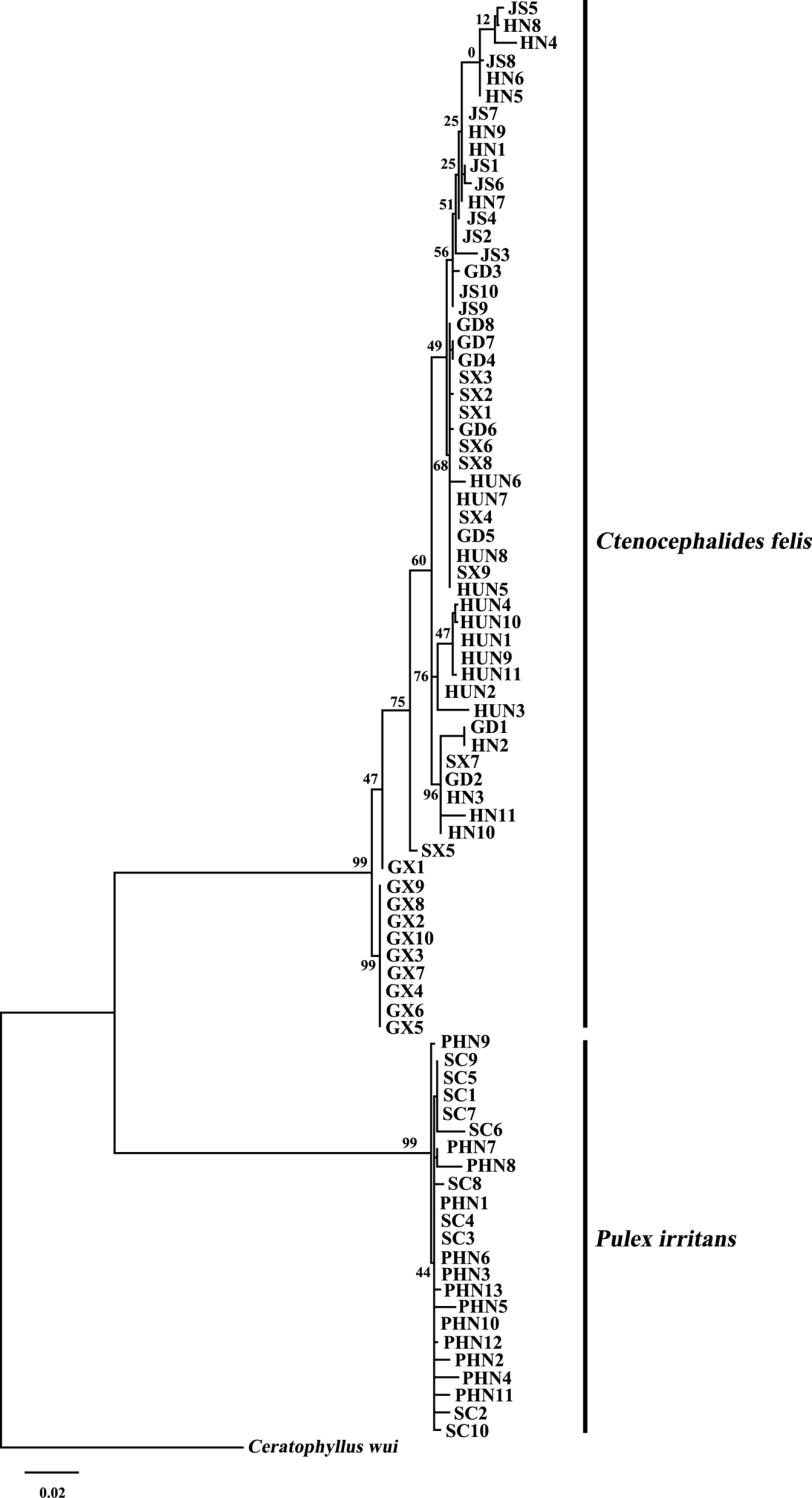
Phylogenetic relationships between *C.felis* and *P.irritans* based on *cox*1 + *cox*2 sequences

The phylogenetic tree constructed based on the EF-1α is shown in Fig. 4, and the phylogenetic tree of the tandem sequences of the *cox*1 and *cox*2 genes is shown in Fig. 5. The *C. felis* and *P. irritans* samples in these two trees were separated with high support. In the evolutionary tree constructed based on EF-1α, *C. felis* was not divided into two distinct branches. However, in the evolutionary tree constructed from the *cox*1 and *cox*2 series, *C. felis* could be divided into two distinct clades; almost all the samples from Guangxi Zhuang Autonomous Region clustered into one branch, and the rest clustered into another. In all of the evolutionary trees, *P. irritans* clustered into a single branch.

## Discussion

Flea is one of the important blood-sucking ectoparasites in veterinary medicine, which can also seriously affect human health. With the development of molecular genetics, the understanding of flea genetic diversity and population dynamics has become the premise for elucidating flea's basic biology and population characteristics, which is of great significance for the detection and control of fleas.

This study showed high genetic diversity based on *cox*1 + *cox*2 sequences of *C. felis* and *P. irritan* from China. The overall genetic diversity of *C. felis* from China was higher than that of Malaysia [12], Thailand, and Fiji and Seychelles [10]. The nucleotide diversity of *cox*1 was higher than that of *cox*2 in *C. felis* and *P. irritans*. This finding is consistent with those of Lawrence et al. [10], who found higher levels of nucleotide diversity in the *cox*1 sequence in the *C. felis* population of Australia and contradicts those of Azrizal-Wahid et al. [12]. The neutral test results of Fu’s Fs and Tajima's D showed that *C. felis* and *P. irritans* in Guangxi Zhuang Autonomous Region and Henan province experienced equilibrium selection and bottleneck effect. Due to long-term interactions between the parasite and the host, certain aspects of the parasite tend to closely track the biological characteristics of the host [47]. Therefore, it stands to reason that the population history of the host may influence the population history of the flea.

*C. felis* and *P. irritan* showed several common haplotypes (A1; A4; B1; B4; AB1; AB5; C1; D1; CD1) and many rare haplotypes (Figs. 2 and 3, Table 4). Multiple *cox*1 gene haplotypes were detected in *C. felis* from Australia, Hong Kong, New Zealand, and Malaysia [12, 29–31], and only one *cox*2 gene haplotype was found in *C. felis* from Australia [30]. However, in our study, the number of *cox*1 and *cox*2 gene haplotypes in *C. felis* was higher than those in the above study. The high genetic diversity of *cox*1 and *cox*2 genes in *C. felis* and *P. irritan* in China may be responsible for many haplotypes. In this study, the most common haplotypes of *C. felis* were A1 haplotype of *cox*1 sequence, B1 haplotype of *cox*2 sequence, and AB1 haplotype of *cox*1 + *cox*2 sequence, while the most common haplotypes of *P. irritan* were C1 haplotype of *cox*1 sequence, D1 haplotype of *cox*2 sequence, and CD1 haplotype of *cox*1 + *cox*2 sequence. This finding suggests that A1, B1, AB1, C1, D1, and CD1 may be the oldest haplotypes because of their higher frequency and wider distribution [48]. At the same time, the Fst values of *C. felis* and *P. irritan* were higher (Fst = 0.34897, Fst = 0.37433), indicating that the genetic differentiation of *C. felis* and *P. irritan* was greater. *Ctenocephalides felis* and *P. irritan* had moderate gene flow (Nm = 0.93, Nm = 0.84), which may result from restricted gene flow between different regions. China is a vast country with complex terrain. Flea transmission between regions may be limited despite well-developed road transportation and frequent human exchanges, resulting in a low distribution of flea haplotypes and limited gene flow.

To further understand the population outcome and genetic differentiation of fleas, *C. felis* and *P. irritan* in this study were compared with previous results. Among them, *C. felis* from Vietnam and Philippines matched their *cox*1 gene with haplotype A4 from Guangxi Zhuang Autonomous Region in this study [32]. The haplotype H1 produced by the *cox*2 gene from Malaysian *C. felis* studied by Azrizal-Wahid et al. [12] was consistent with the haplotype B5 from Guangxi Zhuang Autonomous Region in this study. For *P. irritans*, haplotypes C1 and C10 matched haplotypes H6 and H4 from Spain, respectively [17]. Other haplotypes in this study were not recorded in other countries, indicating that the mutations in our study may not have been dispersed or mutations in other areas have not entered our study area. From the perspective of geographical relationship, southeast Asia is relatively close to Guangxi Zhuang Autonomous Region of China, which may be the reason for the haplotype matching of *C. felis*.

Phylogenetic analysis based on *cox*1 + *cox*2 sequences showed that *C. felis* could be divided into two different branches, and *C. felis* from Guangxi Zhuang Autonomous Region had undergone genetic differentiation. Our study identified two clades of *C. felis* from China through the *cox*1 and *cox*2 genes of mtDNA, indicating two geographical lineages. Only one branch in the evolutionary tree was constructed by EF-1α and *cox*1 + *cox*2 sequences. There were two geographical lineages of *P.irritans* in Spain and Argentina in previous studies, with two possible hidden species [17]. In our study, no multiple lineages of *P.irritans* were found, which may be due to the lack of hidden species of *P.irritans* in China or the limitation of sample size.

Our study showed that *C. felis* populations were geographically isolated, and high levels of genetic diversity and moderate levels of gene flow indicated that *C. felis* and *P.irritans* had undergone genetic differentiation and lineages recombination. *C. felis* may have two genetic lineages, and the Guangxi Zhuang Autonomous Region has undergone genetic differentiation. In contrast, no multiple lineages have been found in human fleas. These results complement previous studies and better understand Chinese fleas population genetics and evolutionary biology.

## Conclusions

In this study, 59 *C. felis* samples and 23 *P. irritans* samples from seven provinces in China were analyzed for population genetic variation and structure using the nuclear genes ITS1 and EF-1α and the mitochondrial genes *cox*1 and *cox*2. The results showed that *C. felis* populations were geographically isolated. The high level of genetic diversity and a moderate level of gene flow indicated that the populations of *C. felis* and *P. irritans* had undergone genetic differentiation and lineage recombination. The results of the neutral test showed that *C. felis* and *P. irritans* in Guangxi Zhuang Autonomous Region and Henan province had experienced equilibrium selection and bottleneck effects. Phylogenetic results indicate that there might be two genetic lineages in *C. felis*, and genetic differentiation has occurred in Guangxi Zhuang Autonomous Region, while no multiple lineages were found in *P. irritans*. These results contribute to a better understanding of fleas' population genetics and evolutionary biology and provide a theoretical basis for further studies.

## Data Availability

The data that support the figures within this paper and other findings of this study are available from the corresponding authors upon reasonable request. All the nucleotide sequences of ITS1, EF-1α, *cox*1, and *cox*2 of *Ctenocephalides felis* and *Pulex irritans* were deposited in GenBank under accession numbers ON113962-ON113964, ON113981-ON114059, ON561113-ON561131, ON569077-ON569087, ON398417-ON398418, ON398481-ON398482, ON398699-ON398702, ON398707-ON398709, ON399054-ON399074, ON399190-ON399208, ON399381-ON399388, ON406172-ON406194, ON455233-ON455234, ON508801-ON508824, ON561108-ON561112.

## Abbreviations

PCR: Polymerase chain reaction
mt: Mitochondrial
ITS1: Internal transcribed spacer 1
ITS2: Internal transcribed spacer 2
EF-1α: Elongation factor 1-alpha
*cox*1: Cytochrome c oxidase subunit 1
*cox*2: Cytochrome c oxidase subunit 2
*cyt*b: Cytochrome b
ML: Maximum likelihood
